# GC-MS and HS-SPME-GC×GC-TOFMS Determination of the Volatile Composition of Essential Oils and Hydrosols (By-Products) from Four *Eucalyptus* Species Cultivated in Tuscany

**DOI:** 10.3390/molecules24020226

**Published:** 2019-01-09

**Authors:** Francesca Ieri, Lorenzo Cecchi, Elena Giannini, Clarissa Clemente, Annalisa Romani

**Affiliations:** 1 PHYTOLAB-DISIA-Department of Informatics, Statistics and Applications “G. Parenti”, University of Florence, Viale Morgagni, 59-50134 Florence, Italy and QuMAP-PIN-Piazza Giovanni Ciardi, 25, 59100 Prato (PO), Italy; francesca.ieri@unifi.it (F.I.); annalisa.romani@unifi.it (A.R.); 2 Department of NEUROFARBA, University of Florence, Viale Pieraccini 6, 50139 Florence, Italy; 3 Versil Green Società Agricola s.s., via dei Cavalli 96, 55054 Massarosa (LU), Italy; info@elenagiannini.com; 4 Department of Pharmacy, University of Pisa, Via Bonanno Pisano 6, 56126 Pisa, Italy; claramente93@hotmail.it

**Keywords:** aromatic water, hydrolat, volatile compounds, metabolic fingerprint, eucalyptol

## Abstract

Essential oils are widely used as functional ingredients for potential multi-purpose functional uses. Hydrosols, co-products of the distillation of plant material, are used in food and cosmetic industries and in biological agriculture, but their volatile composition is poorly investigated. The volatile fractions of essential oils and hydrosols from four less-studied 1,8-cineol-rich *Eucalyptus* species (*E. parvula* L.A.S. Johnson & K.D. Hill, *E. cinerea* F. Muell, *E. pulverulenta* Sims and *E. pulverulenta* baby blue Sims), cultivated in Tuscany in a system of organic farming, were characterized by solvent dilution (essential oils) or extraction (hydrosols) followed by GC-MS and by HS-SPME-GC×GC-TOFMS analysis. GC-MS analysis showed that essential oils were mainly constituted by oxygenated monoterpenes, particularly 1,8-cineole, with monoterpenes hydrocarbons up to 10.8%. Relative differences in the abundance of minor terpenes as limonene, α-pinene, γ-terpinene, *p*-cymene, terpinen-4-ol, α-terpineol, and alloaromandrene were pointed out and seem to be suitable for differentiation among EOs of the four different *Eucalyptus* species. Hydrosols of these species were characterized for the first time: they were mainly constituted by oxygenated monoterpenes (97.6–98.9%), with 1,8-cineole up to 1.6 g/L, while monoterpene and sesquiterpene hydrocarbons were detected only in traces. HS-SPME-GC×GC-TOFMS analysis also allowed providing metabolic profiling of hydrosols for the direct comparison and visualization of volatile components, pointing out the potentially different uses of these products as functional ingredients in food, beverage, and cosmetic industries.

## 1. Introduction

The discovery of the genus *Eucalyptus* (Myrtaceae) came about when James Cook, an explorer, and Sir Joseph Banks, an expert botanist, travelled in Australia in 1770. This genus comprises more than 800 species of native trees and shrubs from Australia belonging to the Myrtaceae family, which are widely grown in many parts of the world [1].

The aromatic volatile oil (essential oil, EO), which is steam-distilled from the foliage, is among the world’s most traded essential oils in terms of volume. The study of EO has attracted much attention for its anti-microbial, antibacterial, antiseptic, fungicidal, and nematicidal activities [2,3,4,5]. EO has a long history of use against the effect of cold, flu, sinusitis, rhinitis and other respiratory infections [6]. Tests in vitro showed that EO from *E. globulus* leaves might be exploited as natural antibiotic for the treatment of several infectious diseases caused by *Escherichia coli* and *Staphylococcus aureus* [7]. Treatment of refrigerated pork with EO led to a significant decrease in *Pseudomonas* spp. count and to an increase of customer acceptance [8]. EO from *E. globulus* and its major compound 1,8-cineole were tested against *A. flavus* and *A. parasiticus*: it was found that the antifungal activity was due not only to the 1,8-cineol, but to the whole phytocomplex [9]. A common need is the availability of natural extracts with pleasant taste and/or smell, combined with a preservative action aimed at avoiding lipid deterioration, fungal growth, oxidation and spoilage by microorganisms. The use of essential oils as functional ingredients in food, beverages and cosmetics is gaining increasing interest because of their relatively safe status, their wide acceptance by consumers, and their exploitation for potential multi-purpose functional use [10,11].

To date, the commercial EOs are mainly obtained from the leaves of the most common species of the genus *Eucalyptus* (i.e., *E. globulus*), which, according to the Standards ISO, must contain 1,8-cineole in percentages higher than 80–85%.

Hydrosols (EW), also known as hydrolats, floral waters, distillate waters or aromatic waters, are the co-products or the by-products of hydro- and steam distillation of plant material. Hydrosols are used in food and cosmetic industries for their organoleptic and biological properties. They are also used in biological agriculture against mushrooms, mildew and insects and for fertilization of soils [12]. Commercial EWs from *Eucalyptus* are currently available in the market, even though their volatile compositions have been poorly investigated to date [13,14]. The major components are generally the same present in oxygenated fraction of the corresponding essential oils [15].

In this study, we took into account four less studied 1,8-cineol-rich *Eucalyptus* species cultivated in Tuscany (central Italy) in a system of organic farming, namely *Eucalyptus parvula* L.A.S. Johnson & K.D. Hill, *Eucalyptus cinerea* F. Muell, *Eucalyptus pulverulenta* Sims and *Eucalyptus pulverulenta* baby blue Sims. The characterization of EOs from *Eucalyptus cinerea* [16,17,18] and *Eucalyptus pulverulenta* [16] has been reported in the literature while, to the authors’ knowledge, no reports on EO from *Eucalyptus parvula* have been published, to date. Essential oil obtained from the leaves of these *Eucalyptus* species could potentially be employed for therapeutic ends and as natural additives for use in the food, cosmetics and perfume industries, extending the use of the plant beyond the predominantly ornamental one.

We aimed to evaluate the content and chemical composition of essential oils (EOs) and, for the first time, leaf hydrosols (EWs) obtained by steam distillation of these species. The evaluation of the content and chemical composition of both EOs and EWs was carried out using optimized Gas-Chromatography coupled with Mass Spectrometry (GC-MS). Head-Space Solid Phase Micro Extraction followed by comprehensive two-dimensional Gas-Chromatography (HS-SPME-GC×GC-TOFMS) analyses allowed providing a fast and direct comparison and visualization of the volatile components (fingerprint) of the EWs, also pointing out the presence of some VOCs not detectable only using the GC-MS. To the author’s knowledge, this work is the first report regarding the characterization of the aroma components of EW from these four *Eucalyptus* species. Due to the fact that these *Eucalyptus* species are 1,8-cineol-rich, we hypothesized that 1,8-cineol was the main volatile of EOs and EWs, and that the main differences among both EOs and EWs from different species were due to relative amounts of minor volatile compounds.

## 2. Results and Discussion

Essential oils and leaf hydrosols were analyzed using integrated sampling and chromatographic techniques. In particular, the volatile organic compounds (VOCs) were extracted (from EWs) or diluted (from OEs) with organic solvent and analyzed by GC-MS and the volatile profile of EWs was also analyzed by GC×GC-TOFMS after extraction of VOCs by HS-SPME. GC-MS is the well-recognized technique of choice for analysis of VOCs from plant material and plant extracts [19,20] and, in this work, we applied this technique for the evaluation of the content and chemical composition of both EWs and EOs. HS-SPME is well recognized as a widespread and convenient sampling tool for VOCs and it is increasingly used coupled with GC-MS in analysis of food and more [21]. However, for quantitation purposes, several issues [22,23] (e.g., differences that arise from different absorption capacity of different fibers, changes in sorption temperature, competition between different molecules at different affinities for the absorpting material, fiber wearing) led to the need of using several devices (e.g., the use of a pool of suitable internal standards [23]) to ensure unbiased quantification. For these reasons, we decided to apply HS-SPME-GC×GC-TOFMS analysis on EWs to better elucidate the volatile profile, thus providing a tool for the direct comparison and visualization of plant volatile components and pointing out the presence of molecules not detectable only with GC-MS. Further quantitative evaluation via HS-SPME-GC-MS analysis can be further investigated in future researches. To the authors’ knowledge, the characterization of EWs of these *E*. species was not been reported in the literature, to date.

### 2.1. Chemical Composition of Essential Oils and Hydrolats by GC-MS

**Figure 1:**
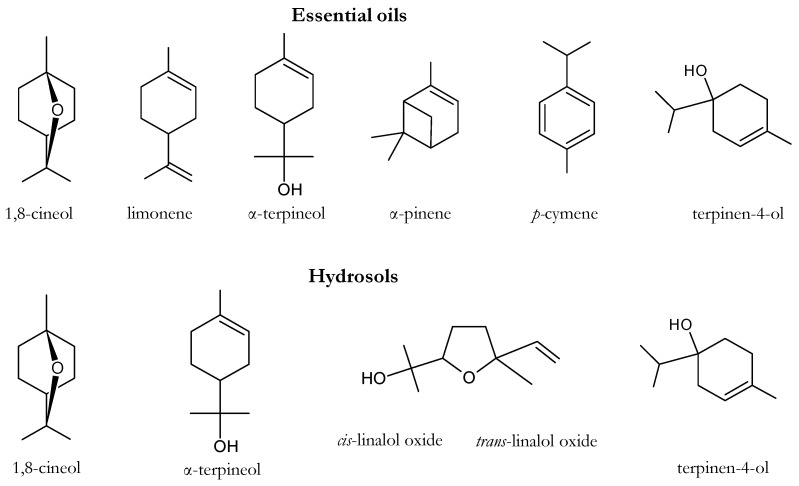
Chemical structure of some of the most abundant molecules in the EWs and EOs.

Table 1 shows the chemical composition by GC-MS of the EOs and EWs summarized in Table 3 (see experimental section). Overall, 10 monoterpene hydrocarbons, 19 oxygenated monoterpenes, 2 sesquiterpene hydrocarbons, 2 aromatic monoterpenes (one of which oxygenated), 1 ester, 4 ketones, 1 aldehyde and 5 alcohols were identified. Some of the main molecules detected in the EWs and EOs are reported in Figure 1. Relative abundance of each of the molecules identified by GC-MS was calculated as a percentage of the peak area on the total area of the identified peaks. Peak areas from the total ion current were normalized by the use of the area of internal standard (tridecane).

Monoterpene hydrocarbons (MH): these terpenes were present in the EO samples with relative abundances between 6.74% and 10.76%. Limonene was the most abundant MH, with similar percentages in all the EOs (3.00–4.65%). Different abundances of the other MHs were pointed out for different *Eucalyptus* species: in *Eucalyptus parvula*, similar amounts of *p*-cymene and α-pinene were detected, followed by lower amounts of γ-terpinene and *Z*-ocimene. In *Eucalyptus cinerea*, similar amounts of α-pinene and Limonene were detected, followed by lower amounts of *p*-cymene, β-myrcene, β-pinene and *Z*-ocimene. Regarding the other two species (*Eucalyptus pulverulenta* Sims and *Eucalyptus pulverulenta* baby blue Sims), the α-pinene amount was approximately half the Limonene, with lower amounts of *p*-cymene, β-myrcene, and β-pinene. Other MH (camphene, α-phellandrene, alloocimene) were in low amounts. Noteworthy, the α-phellandrene content in EOs of all these species was lower than 1%, according to the *European Pharmacopoeia* specification for 1,8-cineol-rich *E*. oils [16].

1,8-cineole (eucalyptol) was by far the main component of the analyzed samples (see the next paragraphs); however, the differences in relative abundance of metabolites present in low amount, or even in trace, play a critical role in mediating different activities for EOs from different *Eucalyptus* species with 1,8-cineole as the main component; indeed, these different activities (i.e., alleophatic [24], protection against *Parthenium hysterophorus* L. [25]) were reported as only due to differences in the relative abundance of minor components [24,25], likely due to the synergistic effect of these latter compounds with other components [26,27].

Regarding the hydrosols, no significant amounts of MH were detected, due to the hydrophobic nature of these molecules.

Oxygenated monoterpenes (OM): 1,8-cineole is the main component of EOs obtained from the leaves of *Eucalyptus globulus,* the most common *Eucalyptus* species [20]. In EOs from these four species, relative abundance of 1,8-cineole ranged between 83.80% and 88.66%, higher than the 80–85% indicated by the standard ISO as the minimum amount of 1,8-cineole for EO from *E. globulus.* Other papers in the literature reported the characterization of EOs from *Eucalyptus cinereal* [16,17,18] and *Eucalyptus pulverulenta* [16], while, to the authors’ knowledge, no reports on EO from *Eucalyptus parvula* have been published, to date. In such papers, the relative abundance of 1,8-cineole showed great variability ranging usually from 58.0 to 69.0% and sometimes reaching 87.8% in *E. cinerea* EOs and being approx. 75% in *E. pulverulenta* EOs. Consequently, also the relative abundances of the other minor terpenes showed a great variability. This variability might be due to the effect of climatic and geographical factors and harvesting season.

In our study, *Eucalyptus parvula* and *Eucalyptus pulverulenta* Sims were the species with the highest amount of 1,8-cineole. Since EOs are totally composed by the volatile fraction, the relative abundance of each compound can be assumed as the amount of this molecule in the oil expressed as g/100g.

Regarding the analyzed hydrosols (EWs), 1,8-cineole was in the range 88.40–90.78%. In order to better characterize these hydrosols, 1,8-cineole was quantified using an external calibration curve, as reported in the experimental section. Table 2 shows that the absolute concentration of 1,8-cineole in the EWs extracts varied in the range 0.74–1.58 g/L, highlighting that this molecule was also recovered in water samples.

In EWs, OMs constituted 98–99% of the total VOCs, according to their water solubility, higher than MHs. Regarding OMs other than 1,8-cineole, in our samples α-terpineol was the most abundant one (4.19–6.24%), followed by lower amounts of terpinen-4-ol, linalool oxides (furanoid, *cis* and *trans*), terpineol isomer, and other minor OMs (<1.5%).

OMs constituted about 90% of the EOs. In these samples, α-terpineol was in the range 2.00–3.11%. Noteworthy, in the EO from *Eucalyptus parvula*, the highest amount of α-terpineol and no presence of its ester, namely α-terpinyl acetate, were detected. In the other three species, α-terpinyl acetate was detected and the sum of the percentages of α-terpineol and α-terpinyl acetate was similar to that of α-terpineol of EO of the *Eucalyptus parvula*. The other OMs didn’t exceed 0.72%.

Other terpenes: no sesquiterpene hydrocarbons were identified in EWs, in agreement with their insolubility in water. In EOs, very low percentages of β-caryophyllene (≤0.07%) in all samples, and slightly higher amounts of alloaromandrene in *Eucalyptus pulverulenta* Sims (0.44%) and *Eucalyptus pulverulenta* baby blue Sims (0.60%) were detected.

One aromatic monoterpene, namely *p*-cymenene, was identified in very low amounts (≤0.04%) only in EOs samples, while one oxygenated aromatic monoterpene (*p*-cymen-8-ol) was identified in low amounts (≤0.11%) only in EWs, according to their different water solubility.

Other compounds: no significant amounts of esters and aldehydes were identified in our samples, the only exceptions being traces of nonanal in one EW sample and very low amounts of isoamyl acetate in EO from *Eucalyptus parvula*. Ketones (the three linear isomers of heptanone and lower percentages of 6-methylhept-5-en-2-one) were identified in low amounts (0.20–0.27% in both EWs and EOs). Finally, alcohols were identified in very low amounts in EOs (heptan-2-ol and heptan-3-ol for a total amount up to 0.16%), while in EWs they were present in percentages up to 2.01%, with 3-methylbutanol as the main molecule, followed by 2-phenylethanol and *Z*-hex-3-en-1-ol.

### 2.2. Fingerprint Analysis by HS-SPME-GC×GC-TOFMS

Solid-phase microextraction (SPME) is a rapid and simple procedure for extraction of volatile fraction from aromatic and medicinal plants [28]. As reported, the divinilbenzene/carboxen/polydimethylsiloxane (DVB/CAR/PDMS) fiber is the most effective SPME fiber able to isolate the volatile fraction from commercial hydrosols of several plants [12]. HS-SPME and GC×GC-TOFMS fingerprint analysis are ideal tools to analyze complex volatile fraction from several matrices, and to provide a sensitive method for the direct comparison and visualization of plant volatile components. As previously reported, 1,8-cineole was the major component in the EOs and EWs, but differences in the other metabolites present in low amounts are very important. Utilization of comprehensive two-dimensional GC (GC×GC) increases separation power with respect to that of the one-dimensional GC in complex matrices where the presence of low abundant components is critical, such as *Eucalyptus* [29]. To our knowledge, there has been no study reporting the volatile profile of EWs from these *Eucalyptus* species; therefore, hydrosols from the four *Eucalyptus* species were analyzed by HS-SPME-GC×GC-TOFMS to better elucidate the volatile profile of these by-products, also pointing out the presence of molecules not detectable with only GC-MS. HS-SPME-GC×GC-TOFMS analyses of the complex volatile fraction of EWs were submitted to advanced fingerprinting analysis of 2D chromatographic data.

**Figure 2:**
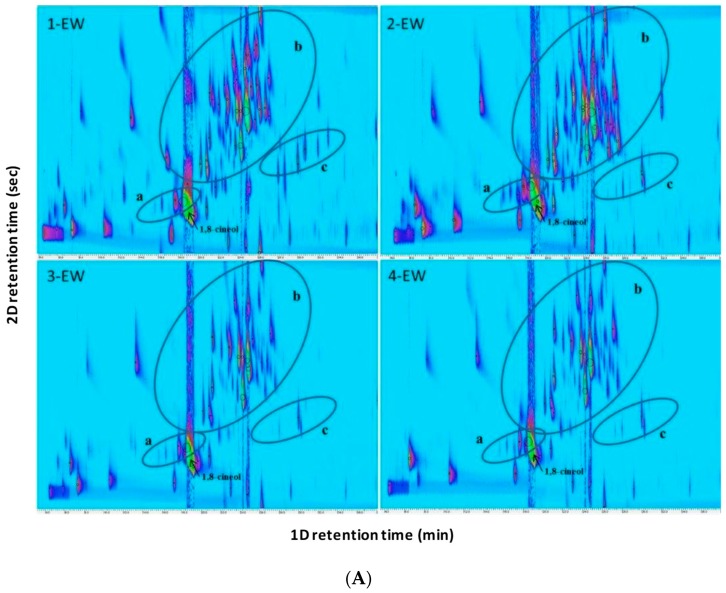

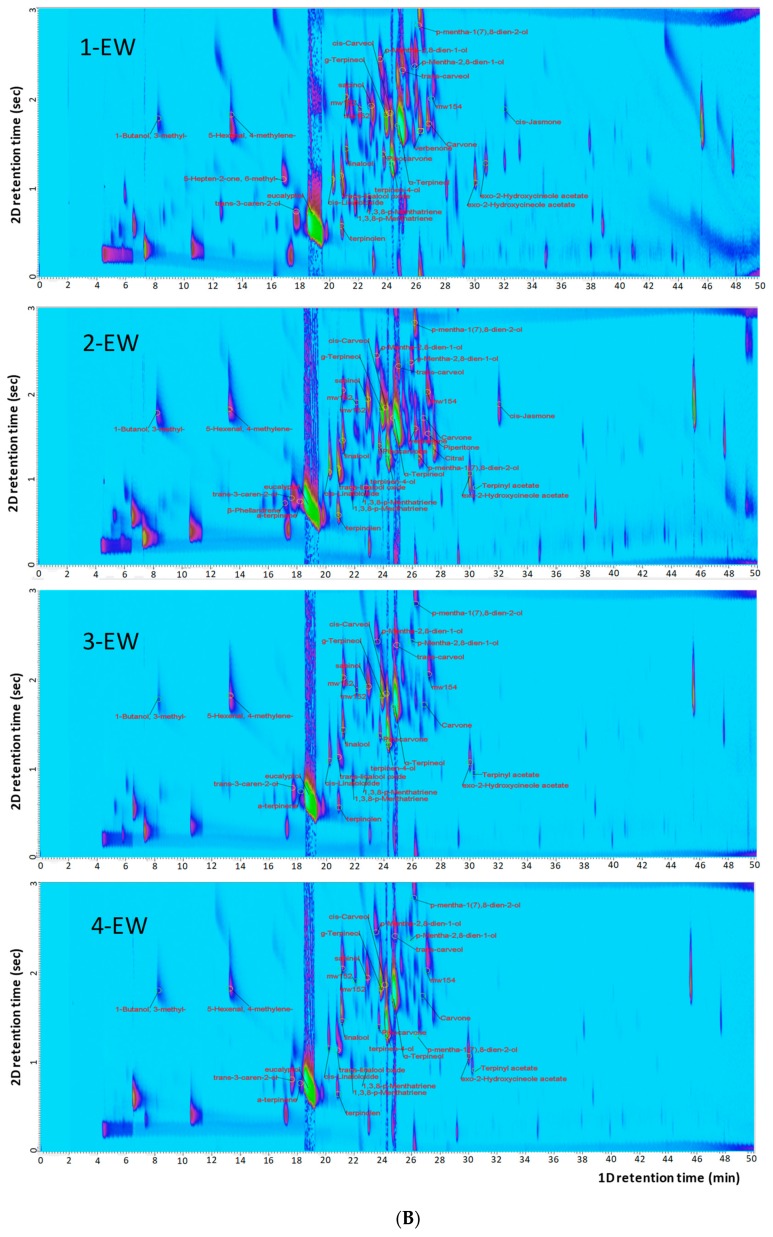
(**A**) 2D contour plots of the analyzed EWs. Braced region a: monoterpenic hydrocarbons; b: oxygenated monoterpenes; c: oxygenated monoterpenes acetate; (**B**) comprehensive template matching fingerprinting with the main identified volatile compounds of 1-16-EW: *E.* parvula L.A.S. Johnson & K.D. Hill; 2-16-EW; *E. cinerea* F. Muell; 3-16-EW: *E. pulverulenta* Sims; 4-16-EW: *E. pulverulenta* baby blue Sims.

In Figure 2, “contour plots” from HS-SPME-GC×GC-TOFMS analyses of the four *Eucalyptus* species are reported: each 2D-peak corresponds to a single volatile compound. In this case, SPME and comprehensive comparative analysis of 2D chromatographic data showed visual differences among EW samples. 1-16-EW and 2-16-EW showed a larger number and a higher intensity of peaks, with respect to 3-16-EW and 4-16-EW. The most intense peak corresponded to 1,8-cineole.

For example, a total of about 400 compounds was detected by GC×GC analysis in 1-16-EW (estimated from the number of peak contours in 2D plots) and, after subtracting baseline peaks, corresponding to fiber blending or background interferences, 137 peaks/compounds were identified. These results were in agreement with Wong et al. [20], where the 2D rational separation pattern aids the identification of ca. 400 metabolites in *Eucalyptus* spp. leaf oils, 183 of which were identified or tentatively identified and represented percentages between 50.8–90.0% of the total ion count, comprising various chemical families.

HS-SPME-GC×GC-TOFMS provided a high metabolic coverage of VOCs: monoterpenes, oxygenated monoterpenes (the main class), oxygenated monoterpenes acetate, and others (ketones, aldehyde, alcohols).

GC×GC is currently adopted as separation technique not only because of its high separation power and sensitivity, but also for its ability to produce more widely distributed and rationalized peak patterns [30] for chemically correlated group of analytes. Terpenic compounds of *Eucalyptus* hydrosols were organized mainly in three clusters in 2D separation space: monoterpenic hydrocarbons, oxygenated monoterpenes and monoterpenes acetate, except for the 1,8-cineol that wrapped around, resulting in monoterpenes zone (Figure 2A). 1,8-cineol showed high secondary retention and fall outside the range of secondary retention time (wrapped around). As previously reported for volatile oil from leaves of *Eucalyptus dunnii* [31], one molecule that wraps around, does not affect the separation and identification of the compounds, since the more strongly retained components (those that wrap-around) did not overlap peaks that were weakly retained in the subsequent modulation.

Up to 31 peaks/compounds belonging to the class of oxygenated monoterpenes were distributed in a defined part of the contour plot for 1-16-EW (Figure 2A, braced region “b”). The number of oxygenated monoterpenes were 33 for 2-16-EW, 23 for 3-16EW and 24 for 4-16-EW.

An advanced approach known as comprehensive template matching fingerprinting [32] was adopted (Figure 2B). This method considers, as a comparative feature, each individual 2D peak together with its time coordinates, detector response and MS fragmentation pattern, and includes them in a sample template that is created by the analyst and can be used to compare plots from different samples directly and comprehensively. A template could be used to correctly interpret visual differences in further analyses. To create the template, the peak identification was performed by matching the experimental mass spectra against spectra databases combined with GC-MS data.

The main differences that emerged between the four varieties could be summarized as follows: 1-16-EW showed the presence of 6-methylhept-5-en-2-one that was not present in the other species (see also Table 1) and the presence of *exo*-2-hydroxycineole acetate isomers. 1-16-EW did not show terpinyl acetate and α-terpinene, which instead were found in the other three species. 2-16-EW was the only species that showed the presence of *β*-phellandrene, piperitone and citral. 1-16-EW and 2-16-EW showed the presence of *cis*-jasmone and carvone, while 3-16-EW and 4-16-EW didn’t show the presence of these molecules. Volatile profiles presented in 2D contour plots allow visual discrimination of the metabolic composition among interspecies of *Eucalyptus* aromatic waters, as reported for leaf oils of other different *Eucalyptus* spp. [20].

## 3. Materials and Methods

### 3.1. Chemicals

All chemicals and standards of analytical reagent grade were from Sigma Aldrich (Steinheim, Germany). Tridecane, 1,8-cineole, heptane and a mixture of linear alkanes (C_10_–C_26_) in hexane were used. Inert gasses (He and N_2_ 99.999% purity) were supplied by SOL gas company (Monza, Italy).

### 3.2. Plant Material

**Figure 3:**
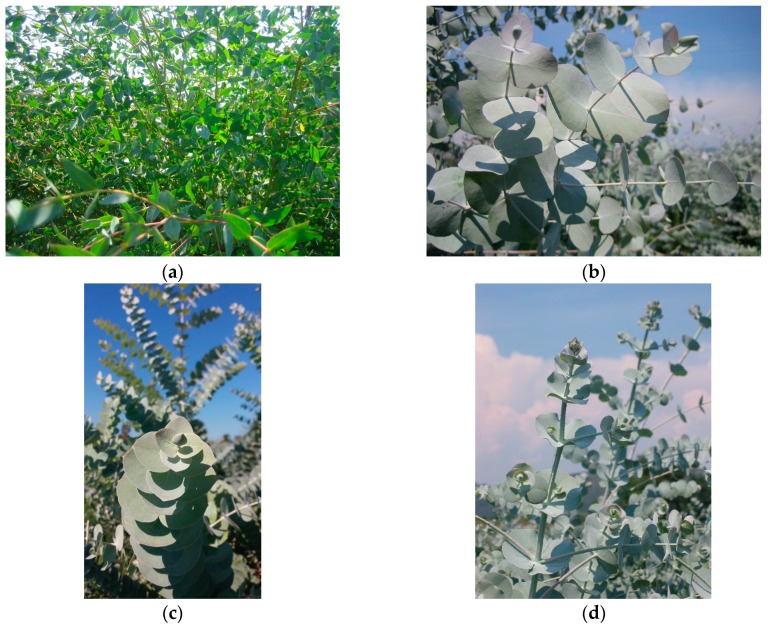
(**a**): *Eucalyptus parvula* L.A.S. Johnson & K.D. Hill; (**b**): *Eucalyptus cinerea* F. Muell; (**c**): *Eucalyptus pulverulenta* Sims; (**d**): *Eucalyptus pulverulenta* baby blue Sims.

In the littoral area of Versilia and Pisa (North-Tuscany-Italy-Latitude: 43.873651; Longitude: 10.328756), Versil Green Società Agricola s.s., a commercial farm, cultivates several species of *Eucalyptus* for the production of ornamental green fronds and essential oils. The cultivated species are: *Eucalyptus parvula* L.A.S. Johnson & K.D. Hill, *Eucalyptus cinerea* F. Muell, *Eucalyptus pulverulenta* Sims and *Eucalyptus pulverulenta* baby blue Sims (Table 3). Figure 3a–d show a picture of each plant. All samples are grown by organic practices, accredited according to the UNI EN 45011 standard. During the production of ornamental fronds, leaves and little stems were separated from the young branches of healthy plants of two years and distilled as reported in Section 3.3.

### 3.3. Obtaining of the Essential Oils and Hydrosols

Essential oils and hydrosols were obtained by steam distillation of the *eucalyptus* fresh leaves and little stems, within 24 h after harvesting, using the Essenziale 20 extractor (Tred Technology srl, Italy). The system used low working temperatures (always below 80 °C), thus decreasing the consumption energy and minimizing the degradation of the volatile fraction. The process parameters were continuously checked and adjusted during the distillation, e.g., internal pressure inside the boiler, internal boiler temperature, water temperature, oil and hydrosol flow. The starting material vs hydrosol ratio was 3:1 and the recovery of the corresponding essential oil was in variable percentage depending on the collection period and atmospheric conditions. The mean of yields of EO are from 1.1 to 1.3%, as reported in Table 3.

### 3.4. Analysis of Essential Oils and Aromatic Waters

#### 3.4.1. GC-MS Analysis

EO samples were diluted 10,000 times with heptane, in presence of tridecane (20 ppm), for avoiding saturated signals during the following chromatographic analysis.

Regarding EWs, 0.5 mL of sample were extracted with 0.5 mL of heptane (with 20 ppm, tridecane) for 1 h with an automatic stirrer; water residues were removed using anhydrous sodium sulfate and the obtained organic extracts were diluted 20 times with heptane.

GC-MS analysis of EO and EW solutions, obtained as described above, were carried out by liquid injection on an Agilent 7890a Gas Chromatograph equipped with a Gerstel MPS automatic sampler system and a quadrupole Mass Spectrometer 5975c MSD (Agilent Technologies, Palo Alto, CA, USA) working in split-less mode. The analytes separation was carried out by an Agilent DB InnoWAX column (length, 50 m; i.d., 200 µm, film thickness, 0.4 µm). Initial oven temperature was 40 °C, held for 1 min. Then, it raised to 200 °C at 5 °C min^−1^, then raised to 260 °C at 10 °C min^−1^ and finally held at 260 °C for 6 min. Injector temperature was 260 °C, while the carrier gas helium, was at a flow rate of 1.2 mL/min. 1 µL of each sample was injected.

Mass spectrometer worked in the mass range 40–350 *m*/*z* and with an electron ionization of 70 eV and the Total Ion Current chromatograms were recorded. Compounds were tentatively identified by comparing the mass spectra of each peak with those reported in mass spectral database as the standard NIST08/Wiley98 libraries; when available, standards were used for confirming the nature of the identified molecules: α-pinene, β-pinene, camphene, β-myrcene, α-phellandrene, limonene, *Z*-ocimene, γ-terpinene, *p*-cymene, alloocimene, 1,8-cineol, linalool, terpinen-4-ol, citral, α-terpineol, borneol, β-caryophillene, heptan-2-one, 6-methylhept-5-en-2-one, nonanale, 3-methylbytanol, heptan-2-ol and 2-phenylethanol. Peaks identification was then confirmed by comparing their retention index; to this aim, a mixture of linear alkanes (C_10_–C_26_) in hexane (Sigma Aldrich, Saint Louis, MI, USA) was injected in the same condition already described for sample analysis and the retention indexes were calculated by the generalized equation [33] and compared with the literature [34] The relative concentration of each identified compound was calculated as peak area on total area of all the identified peaks (peaks areas were normalized using tridecane as internal standard). 1,8 cineole in EWs was quantified by a six point calibration curve, which were built using 1,8 cineole as external standard (range 10–160 ppm, 0.9936 R^2^).

#### 3.4.2. HS-SPME-GC×GC-TOFMS Analysis

The EWs from the four *Eucalyptus* species (Table 3) were extracted by solid-phase microextraction (SPME) and analyzed by GC×GC-TOFMS. GC×GC was performed by a flow modulation apparatus consisting on an Agilent 7890B GC (Agilent Technologies, Palo Alto, CA, USA), with flow modulator device for 2D separation, coupled with a time-of-flight mass spectrometer (TOF-DS Markes International Ltd., Llantrisant, UK). After some trials aimed at optimizing amounts of sample, NaCl and water and exposure time and temperature, SPME conditions were set as follow: 1 mL of the EW sample, together with 2 g of NaCl and 4 mL of deionized water were placed into a 20-mL screw cap vial fitted with PTFE/silicone septa. VOCs were absorbed exposing a divinilbenzene/carboxen/polydimethylsiloxane (DVB/CAR/PDMS) 2 cm fiber (Supelco) for 10 min into the vial at 60 °C and then immediately desorbed at 280 °C in a gas chromatograph injection port.

Chromatographic separation was performed using a first dimension (^1^D) HP-5 column (20 m × 0.18 mm I.D. × 0.18 μm film thickness (*df*); Agilent Technologies, Palo Alto, CA, USA), and a WAX second dimension (^2^D) column (5 m × 0.32 mm I.D. × 0.15 μm *df*; Agilent Technologies, Palo Alto, CA, USA).

Flow modulation was performed with a modulation period of 3 s. Helium was used as carrier gas (99.999% purity) at flow rates of 0.4 and 10 mL/min in first and second dimensions, respectively.

The chromatographic conditions were: oven temperature program, 40 °C, increased at 4 °C/min to 220 °C, increased at 10 °C/min to 260 °C (hold 1 min); injector temperature, 260 °C; split ratio 1:5. The inlet of the ^2^D column was maintained under vacuum by a deactivated fused silica (0.30 m × 0.10 mm I.D.) placed immediately before the column, after the flow modulator. TOFMS parameters: the ion source temperature was 230 °C; the transfer line temperature was 280 °C; ionization, −70 eV. A mass range of 43–500 Da was used, with data rate of 50 Hz. TOF-DS ^TM^ software, version 2.0 (Markes International Ltd.; Llantrisant, UK, 2016) was used for data acquisition. GC IMAGE version R2.5 GC×GC (64 bit) software (GC IMAGE; LCC-Lincon, NE, USA, 2014) was used for data processing.

### 3.5. Statistical Analysis

The semi-quantitative (Table 1) and quantitative data (Table 2) are expressed as the mean of three determinations. Statistical significance was evaluate applying one-way ANOVA and F-test (*p* < 0.05) using Microsoft Excel statistical software; means were then compared by Fisher’s LSD test using the DSAASTAT excel^®^ VBA macro, version 1.1 (Onofri, A.; Pisa, Italy, 2007).

## 4. Conclusions

This study reports a first preliminary characterization of the volatile profile of EO and EW of four less studied 1,8-cineol-rich *Eucalyptus* species (*E. parvula* L.A.S. Johnson & K.D. Hill, *E. cinerea* F. Muell, *E. pulverulenta* Sims and *E. pulverulenta* baby blue Sims) cultivated in Tuscany (Italy) and intended to be employed as natural additives in the food, cosmetics and perfume industries, as well as for therapeutic ends, beyond the predominantly ornamental one. Chemical differences in VOCs from EWs and EOs were evidenced, providing products for potentially different uses. Further studies will be necessary for the standardization of commercial EOs and EWs optimizing the technological harvesting period.

Regarding the EOs from these *Eucalyptus* species, GC-MS analysis of diluted samples showed that oxygenated monoterpenes accounted for up to 92.7% and monoterpenes hydrocarbons contributed to volatile fraction for up to 10.8%, with limonene as the most representative MH. The relative abundance of minor terpenes as limonene, α-pinene, γ-terpinene, *p*-cymene, terpinen-4-ol, α-terpineol and alloaromandrene seems to be suitable for differentiation among EOs of the four different *Eucalyptus* species.

GC-MS analysis allowed pointing out that the volatile fraction of EWs extracts was mainly constituted by oxygenated monoterpenes (97.6–98.9%), with monoterpene and sesquiterpene hydrocarbons detected only in traces. HS-SPME-GC×GC-TOFMS analysis of EWs extracts also allowed for metabolic profiling of EWs for the direct comparison and visualization of volatile components of these not yet investigated co-products. The GC-MS quantitative evaluation of 1,8-cineole in EWs showed amounts of up to 1.6 g/L; consequently, the studied EWs, co-products of steam distillation of fresh leaves and little stems of the four *Eucalyptus* species, can be proposed as functional ingredients for the food, beverage, and cosmetic industries.

## Figures and Tables

**Table 1:** Volatile organic compounds in hydrosols (EW) and essential oils (EO) of four *Eucalyptus* species identified by liquid injection GC-MS analysis as described in paragraph 3.4.1. For each compound, concentration is expressed as area % on the total area after normalization with ISTD. Data are the mean of three determinations. Retention Indices (RI_cal_): Non-isothermal Kovats retention indices from temperature-programming, using the definition of Van den Dool and Kratz, 1963. Retention Indices (RI_ref_): Non-isothermal Kovats retention indices from temperature-programming from Chemistry WebBook. For each compound, different letters indicate significant differences by Fisher’s LSD test (z, y, x, w for aromatic waters; a, b, c, d, e for essential oils).

n°	Compound	RI_cal_	RI_ref_	Hydrosols (Area %)						Essential Oils (Area %)					
				1-16-EW	2-16-EW	3-16-EW	4-16-EW	1-17-EW	1-1617-EW	1-16-EO	2-16-EO	3-16-EO	4-16-EO	1-17-EO	1-1617-EO
	**Monoterpene Hydrocarbons**			**0.22**	**0.02**	**0.00**	**0.02**	**0.01**	**0.00**	**8.05**	**10.76**	**7.10**	**8.34**	**6.74**	**7.54**
1	α-pinene	1030	1026	0.09	-	-	-	-	-	1.20 b	4.45 e	2.38 d	2.15 c	0.95 a	1.12 ab
2	camphene	1067	1065	-	-	-	-	-	-	0.03 bc	0.04 c	0.02 ab	0.02 ab	0.01 a	0.02 ab
3	β-pinene	1122	1118	-	-	-	-	-	-	0.11 ab	0.17 cd	0.14 bc	0.19 d	0.09 a	0.10 a
7	β-myrcene	1168	1167	-	-	-	tr	tr	-	0.12 a	0.22 c	0.17 b	0.25 c	0.09 a	0.11 a
8	α-phellandrene	1177	1177	-	-	-	-	-	-	0.15 b	0.13 b	0.04 a	0.01 a	0.15 b	0.14 b
12	Limonene	1214	1210	0.08 y	0.02 z	tr	0.02 z	0.01 z	-	3.60 b	4.65 d	3.66 b	4.29 c	3.00 a	3.41 b
14	*Z*-ocimene	1241	1242	-	-	-	-	-	-	0.36 c	0.17 b	0.07 a	0.07 a	0.45 d	0.39 c
15	γ-terpinene	1259	1254	tr	-	-	-	-	Tr	0.54 b	0.13 a	0.10 a	0.07 a	0.48b	0.50 b
16	*p*-cymene	1286	1281	0.05	-	-	tr	tr	-	1.78 e	0.73 b	0.50 a	1.23 c	1.42 cd	1.63 de
20	alloocimene	1383	1377	-	-	-	-	-	-	0.15 d	0.07 b	0.03 a	0.04 a	0.12 c	0.13 c
	**Oxygenated Monoterpenes**			**98.62**	**98.50**	**98.29**	**97.64**	**98.91**	**98.80**	**91.30**	**88.74**	**91.99**	**90.60**	**92.71**	**91.89**
11	2,3-dehydro-1,8-cineole	1202	1197	-	-	-	-	-	-	-	0.09 c	0.08 bc	0.07 b	0.05 a	0.05 a
13	1,8-cineol	1225	1221	89.53 y	88.40 z	90.78 x	89.17 zy	90.22 yx	89.35 zy	87.06 b	83.80 a	87.72 bc	85.14 a	88.66 c	87.75 bc
23	*cis*-linalool oxide (furanoid)	1453	1453	0.5 zy	0.77 x	0.47 zy	0.44 z	0.52 zy	0.55 y	-	-	-	-	-	-
25	*trans*-linalool oxide (furanoid)	1481	1482	0.49 y	0.68 x	0.36 z	0.43 zy	0.46 zy	0.52 y	-	-	-	-	-	-
26	linalool	1545	1544	-	-	-	-	-	-	0.05 b	0.09 c	0.09 c	0.11 d	0.02 a	0.03 a
27	fenchyl alcohol	1595	1571	tr	tr	-	tr	tr	-	0.06 b	0.08 c	0.05 b	0.06 b	0.06 b	0.03 a
28	pinocarvone	1600	1575	-	-	-	-	-	-	0.06 b	0.03 a	0.05 b	0.06 b	0.06 b	0.05 b
29	terpinen-4-ol	1617	1612	0.92 y	0.94 yx	1.01 x	1.37 w	0.75 z	0.87 y	0.51 cd	0.47 bc	0.56 d	0.72 e	0.39 a	0.44 b
31	*cis*-*p*-mentha-2,8-dienol	1637	1642	0.07 zy	0.06 z	0.12 x	0.08 y	0.12 x	0.08 y	0.02 a	0.03 ab	0.06 c	0.08 d	0.04 b	0.04 b
33	*trans*-pinocarveol	1683	1659	0.17 z	0.24 yx	0.23 yx	0.24 yx	0.25 x	0.21 y	0.09 ab	0.13 c	0.11 bc	0.08 a	0.10 ab	0.09 ab
34	*trans*-*p*-mentha-2,8-dienol	1679	1670	0.11 z	0.10 z	0.11 z	0.12 z	0.13 z	0.10 z	-	-	-	-	-	-
35	terpineol isomer	1682	-	0.25 z	0.40 y	0.37 y	0.42 y	0.25 z	0.24 z	0.14 ab	0.19 c	0.17 bc	0.23 d	0.12 a	0.14 ab
36	citral	1698	1695	0.17 x	0.27 w	0.19 x	0.10 z	0.14 y	0.19 x	0.05 ab	0.07 bc	0.08 c	0.05 ab	0.04 a	0.05 ab
37	α-terpineol	1707	1704	6.02 x	6.24 x	4.19 z	4.50 z	5.53 y	6.21 x	3.11 c	2.77 b	2.00 a	2.20 a	3.02 bc	3.08 bc
38	borneol	1718	1715	tr	0.05 x	-	0.04 y	0.02 z	-	-	-	-	-	-	-
39	α-terpinyl acetate	1722	1721	-	-	-	-	-	-	0.02 a	0.90 b	0.92 b	1.71 c	0.02 a	-
40	*cis*-carveol	1850	1847	0.14 yx	0.11 z	0.12 zy	0.10 z	0.18 w	0.16 xw	-	-	-	-	-	-
42	*exo*-2-hydroxycineole	1870	1870	0.08 z	0.13 y	0.21 x	0.52 w	0.10 zy	0.11 zy	-	-	-	-	-	-
43	*cis*-*p*-mentha-1(7),8-dien-2-ol	1905	1888	0.17 y	0.11 z	0.13 z	0.11 z	0.24 w	0.21 x	0.12 bc	0.09 ab	0.09 ab	0.07 a	0.13 c	0.14 c
	**Sesquiterpene Hydrocarbons**			-	-	-	-	-	-	0.09	0.10	0.47	0.63	0.02	0.03
30	β-caryophyllene	1631	1625	-	-	-	-	-	-	0.07 b	0.07 b	0.03 a	0.03 a	0.02 a	0.03 a
32	alloaromandrene	1640	1645	-	-	-	-	-	-	0.02 a	0.02 a	0.44 b	0.60 c	-	-
	**Aromatic Monoterpenes**			-	-	-	-	-	-	0.03	0.04	0.02	0.03	0.04	0.03
24	*p*-cymenene	1455	1455	-	-	-	-	-	-	0.03 b	0.04 c	0.02 a	0.03 b	0.04 c	0.03 b
	**Oxygenated Aromatic Monoterpenes**			0.06	0.11	0.08	0.10	0.10	0.08	-	-	-	-	-	-
41	*p*-cymen-8-ol	1857	1869	0.06 z	0.11 x	0.08 zy	0.10 yx	0.10 yx	0.08 zy	-	-	-	-	-	-
	**Ester**			-	-	-	-	-	-	0.04	-	-	-	0.03	0.05
4	isoamyl acetate	1125	1126	-	-	-	-	-	-	0.04 a	-	-	-	0.03 a	0.05 a
	**Ketones**			0.21	0.22	0.20	0.23	0.20	0.27	0.24	0.20	0.25	0.24	0.22	0.22
5	heptan-4-one	1132	-	-	-	-	-	-	-	0.06 c	0.03 a	0.04 ab	0.04 ab	0.04 ab	0.05 bc
6	heptan-3-one	1161	1163	0.05 z	0.06 zy	0.06 zy	0.07 yx	0.05 z	0.08 x	0.07 a	0.06 a	0.07 a	0.06 a	0.06 a	0.07 a
9	heptan-2-one	1190	1185	0.12 z	0.16 z	0.14 z	0.16 z	0.13 z	0.16 z	0.12 a	0.11 a	0.14 a	0.14 a	0.12 a	0.11 a
19	6-methylhept-5-en-2-one	1346	1338	0.04 x	-	-	-	0.02 z	0.03 y	tr	-	-	-	-	-
	**aldehyde**			-	-	tr	-	-	-	-	-	-	-	-	-
22	nonanal	1405	1401	-	-	tr	-	-	-	-	-	-	-	-	-
	**Alcohols**			0.89	1.15	1.43	2.01	0.78	0.85	0.14	0.14	0.15	0.14	0.14	0.16
10	3-methylbutanol	1198	1210	0.46 z	0.76 y	0.68 y	1.07 x	0.45 z	0.49 z	-	-	-	-	-	-
17	heptan-3-ol	1290	-	0.07 zy	0.08 yx	0.06 z	0.08 yx	0.06 z	0.09 x	0.07 a	0.06 a	0.08 a	0.07 a	0.06 a	0.08 a
18	heptan-2-ol	1314	1318	0.06 z	0.09 x	0.08 yx	0.09 x	0.07 zy	0.08 yx	0.07 a	0.08 a	0.08 a	0.07 a	0.07 a	0.08 a
21	*Z*-hex-3-en-1-ol	1384	1384	0.14 x	0.07 z	0.09 zy	0.24 w	0.12 yx	0.13 yx	-	-	-	-	-	-
44	2-phenylethanol	1928	1924	0.16 y	0.15 y	0.52 x	0.53 x	0.08 z	0.06 z	-	-	-	-	-	-

**Table 2:** Content of 1,8-cineole in the hydrosols by GC-MS analysis. Data are expressed in g/L as mean of three independent determinations (SD < 3%). Different letters indicate significant differences at *p* < 0.05.

Sample	Kind of Sample	1,8-cineole (g/L)
1-16-EW	hydrosol	1.58 a
2-16-EW	hydrosol	1.45 b
3-16-EW	hydrosol	1.52 a
4-16-EW	hydrosol	0.74 e
1-17-EW	hydrosol	0.86 d
1-1617-EW	hydrosol	1.20 c

**Table 3:** List of the analyzed samples. EW: hydrosol or aromatic water; EO: essential oil. 1, *E. parvula* L.A.S. Johnson & K.D. Hill; 2, *E. cinerea* F. Muell; 3, *E. pulverulenta* Sims; 4, *E. pulverulenta* baby blue Sims. 16 and 17 indicate the year in which the sample was obtained. 1617 indicates samples obtained as a mixture in equal parts of samples from 2016 and 2017.

Sample Name	Kind of Sample	Eucalyptus Species	Year	Yields %
1-16-EW	aromatic water	*E. parvula* L.A.S. Johnson & K.D. Hill	2016	
2-16-EW	aromatic water	*E. cinerea* F. Muell	2016	
3-16-EW	aromatic water	*E. pulverulenta* Sims	2016	
4-16-EW	aromatic water	*E. pulverulenta* baby blue Sims	2016	
1-17-EW	aromatic water	*E. parvula* L.A.S. Johnson & K.D. Hill	2017	
1-1617-EW	aromatic water	*E. parvula* L.A.S. Johnson & K.D. Hill	2016–2017	
1-16-EO	essential oil	*E. parvula* L.A.S. Johnson & K.D. Hill	2016	1.2
2-16-EO	essential oil	*E. cinerea* F. Muell	2016	1.1
3-16-EO	essential oil	*E. pulverulenta* Sims	2016	1.1
4-16-EO	essential oil	*E. pulverulenta* baby blue Sims	2016	1.1
1-17-EO	essential oil	*E. parvula* L.A.S. Johnson & K.D. Hill	2017	1.3
1-1617-EO	essential oil	*E. parvula* L.A.S. Johnson & K.D. Hill	2016–2017	
